# Development of Rapid Detection and Genetic Characterization of *Salmonella* in Poultry Breeder Feeds

**DOI:** 10.3390/s90705308

**Published:** 2009-07-06

**Authors:** Robin Jarquin, Irene Hanning, Soohyoun Ahn, Steven C. Ricke

**Affiliations:** 1 Dept. of Poultry Science, University of Arkansas, Fayetteville, AR 72704, USA; E-Mail: robin.jarquin@cobb-vantress.com; 2 Research and Development, Cobb-Vantress Incorporated, P.O. BOX 1030, Siloam Springs, AR 72761, USA; 3 Dept. of Food Science, University of Arkansas, Fayetteville, AR 72704, USA; E-Mail: ihanning@uark.edu; 4 Food Science and Technology Program, Arkansas State University, State University, AR 72467, USA; E-Mail: sahn@astate.edu

**Keywords:** *Salmonella*, poultry, breeder, feed, biosensors, detection

## Abstract

*Salmonella* is a leading cause of foodborne illness in the United States, with poultry and poultry products being a primary source of infection to humans. Poultry may carry some *Salmonella* serovars without any signs or symptoms of disease and without causing any adverse effects to the health of the bird. *Salmonella* may be introduced to a flock by multiple environmental sources, but poultry feed is suspected to be a leading source. Detecting *Salmonella* in feed can be challenging because low levels of the bacteria may not be recovered using traditional culturing techniques. Numerous detection methodologies have been examined over the years for quantifying *Salmonella* in feeds and many have proven to be effective for *Salmonella* isolation and detection in a variety of feeds. However, given the potential need for increased detection sensitivity, molecular detection technologies may the best candidate for developing rapid sensitive methods for identifying small numbers of *Salmonella* in the background of large volumes of feed. Several studies have been done using polymerase chain reaction (PCR) assays and commercial kits to detect *Salmonella* spp. in a wide variety of feed sources. In addition, DNA array technology has recently been utilized to track the dissemination of a specific *Salmonella* serotype in feed mills. This review will discuss the processing of feeds and potential points in the process that may introduce *Salmonella* contamination to the feed. Detection methods currently used and the need for advances in these methods also will be discussed. Finally, implementation of rapid detection for optimizing control methods to prevent and remove any *Salmonella* contamination of feeds will be considered.

## 1. Introduction

*Salmonella* is the leading cause of foodborne illness in the United States. In 29% of *Salmonella* infections, or approximately 406,000 cases annually, poultry has been identified as the primary source of infection [1,2]. The costs associated with non-typhoidal *Salmonella* infections are estimated at nearly $2.4 billion dollars annually, which includes costs due to loss of productivity and medical treatment costs. A poultry producer suffers losses due to *Salmonella* infection of the flock including loss of birds and production time. These losses in the United States per year have been calculated to be approximately $64 million – $114 million, but these calculated losses do not take into account the loss of eggs and other consumable poultry products.

The use of antibiotics to control *Salmonella* in poultry is not an option and alternatives to antibiotics for control of bacteria in poultry including bacteriophage and probiotics have yet to be completely successful. If one bird in a flock becomes infected with *Salmonella*, the infection can spread rapidly and the entire flock can become infected within 2 to 10 days. Since *Salmonella* may remain in the environment between flocks, control of infection initially can help reduce and eliminate environmental contamination. Hence, constant monitoring and rapid detection are needed to prevent *Salmonella* infection in poultry flocks.

*Salmonella* may be introduced to a flock by multiple environmental sources, but poultry feed is suspected to be a leading source. Detecting *Salmonella* in feed can be challenging because low levels of the bacteria may not be recovered using traditional culturing techniques. Numerous detection methodologies have been examined over the years for quantifying *Salmonella* in feeds and some have proven to be more effective for *Salmonella* isolation and detection in a variety of feeds. However given the potential need for increased detection sensitivity, molecular detection technologies may be the best candidate for developing rapid sensitive methods for identifying small numbers of *Salmonella* in the background of large volumes of feed. The primary difficulty with routine application of molecular assays is the problem of extracting and recovering representative samples from feeds for molecular analyses. Molecular techniques also may be hindered due to chemicals present in feed samples that can inhibit PCR reactions. This review will discuss the processing of feeds and potential points in the process that may introduce *Salmonella* contamination to the feed. Detection methods currently used and the need for advances in these methods also will be discussed. Bead-based DNA arrays for simultaneous detection of multiple *Salmonella* serotypes offer new possibilities for rapid detection and these innovations are presented. Finally, implementation of rapid detection for optimizing control methods to prevent and remove any *Salmonella* contamination of feeds will be considered.

## 2. *Salmonella* in Broiler Breeders

**Figure 1.**
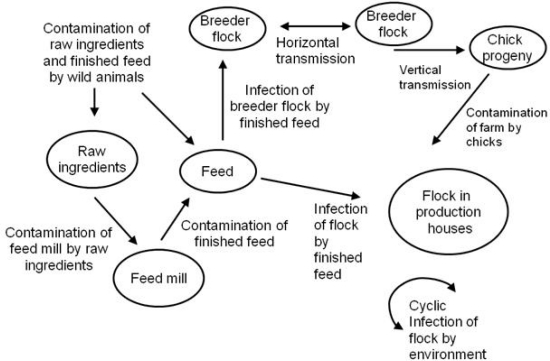
A diagram of the possible routes of dissemination of *Salmonella* from broiler breeder flocks to farm environments and possible routes of persistence.

It has long been recognized that breeding stock of poultry play a crucial role in controlling the dissemination of *Salmonella* infection and contamination [3,4]. Young chicks in the hatchery are more susceptible to infection with *Salmonella* due to an absence of protective gut microflora. For this reason, 1-day old chicks can be colonized with as few as 5 cells of *Salmonella*, but colonization of 2 week old birds which have protective microflora is inconsistent and requires higher doses [5]. Furthermore, the susceptibility of these young chicks results in rapid horizontal transmission [6]. Surveys and estimates of salmonellae-positive chicks leaving the hatchery range from 4.8 to 9% [7,8]. The dissemination of *Salmonella* from broiler breeder flocks to farm environments and possible routes of persistence are diagramed in Figure 1.

Infected chicks from a hatchery that are placed on a grow-out farm can act as sources of infection and contamination to the farm environment [9]. *Salmonella* has been demonstrated to persist in farm environments for 1 year with or without poultry being present [10]. Furthermore, total disinfection of grow-out farms may be impossible to achieve due to cleaning difficulties and environmental reservoirs such as mice and wild birds [11].

*Salmonella* contamination on the broiler grow-out farms is complex and can come from multiple sources in the environment such as feed, feed ingredients, water, litter and from breeding stock [6,7,12–16]. However, it is more difficult to determine the sources of Salmonella to primary broiler breeder flocks. Primary breeding flocks are substantially more valuable than other poultry stock and therefore the hatchery design is usually state-of-the-art with a one-way movement of clean to dirty flow design to reduce contamination. Incidences are typically lower in broiler breeder hatcheries [17]. These breeder flocks are much smaller and hatching eggs are gathered more frequently and disinfected shortly after being gathered [4].

Feed has been implicated as an important source of *Salmonella* to poultry [18,19]. Hinton [20] demonstrated that *Salmonella* infection could become established in day old chicks fed 0.1 to 0.3 cells of *Salmonella* per gram of feed. Modern culturing techniques require enrichment in order to detect such a low number of cells and molecular techniques are not sensitive enough to detect such low numbers. For this reason, some *Salmonella* contamination of feed may pass undetected. Sources of *Salmonella* and the processing of feed will be discussed in the next section.

## 3. Feed

It has been suggested that occurrence of *Salmonella* contamination in feeds produced in feed mills may be due to transfer of *Salmonella* from birds, rodents or other pests [21]. In addition, contamination of feed mill ingredient intake pits and outloading gantries for finished feed products by wild-bird droppings containing *Salmonella* has been described [22]. Pelleted and mash poultry feeds have long been recognized as vectors for *Salmonella* contamination in poultry production systems with ingredients of animal origin having the highest frequencies of contamination [23,24]. However, ingredients of vegetable origin also have been reported to harbor the organism [18]. Since animal feed is the first portion of the farm to fork continuum for food safety, it represents a critical point for intervention and control of *Salmonella*.

### 3.1. Sources of Salmonella to Feed

Morris *et al*. [25] found that of all the samples taken from a commercial broiler operation, feed samples were most frequently contaminated with *Salmonella*. Human outbreaks of Salmonellosis have been traced back to feed for decades. In 1958, an outbreak of infection of *S.* Hadar in Israel was linked to the consumption of chicken liver and was eventually traced back to bone meal fed to the chickens [26]. Frozen chickens from a packing plant in Cheshire, England, were implicated in a large outbreak in 1968 of infection with *S.* Virchow [27]. The investigation showed that the hatchery and some rearing farms that supplied the packing plant contained chickens colonized with *S.* Virchow. In this investigation, the same serotype of *Salmonella* was isolated from feed fed to the chickens [28]. Chickens served in a restaurant in Arkansas caused an outbreak of *S.* Agona. The chickens were traced to a farm in Mississippi that fed the chickens with feed containing Peruvian fish meal found to be contaminated with *S.* Agona [29] The fish meal was found to be the ultimate source for a number of *S.* Agona infections in the United States, the United Kingdom, Israel, and the Netherlands.

Several more recent investigations using sophisticated genotyping methods have found confirmed that *Salmonella* in feed can be a primary source of contamination. In a study by Shirota *et al*. [30], *S.* Enteritidis strains obtained from feed samples and egg contents taken from a layer farm showed pulsed-field gel electrophoresis (PFGE) patterns that were genetically related. Futhermore, the isolates belonged to a single phage type which suggested that the contamination of the farms was linked to the occurrence of salmonellae in feed. Using PFGE, Wasyl *et al*. [31] found *Salmonella* isolates with identical pulse-types isolated from feed and poultry. Bucher *et al*. [32] also used PFGE along with serotyping, phage typing and antimicrobial resistance typing and concluded that *Salmonella* strains isolated from broiler feed were indistinguishable from strains isolated in packaged raw, frozen chicken nuggets and strips.

### 3.2. Processing of Feed

Feed is typically comprised of corn, soybeans, oats, alfalfa, calcium and a vitamin mixture [33]. This composition may vary depending on the manufacturer and the type of poultry being fed. For example, laying hens require higher concentrations of calcium for egg shell production. To produce the feed, ingredients are mixed and steam processed. After processing, the feed may be cooled by passing through a cooling air unit or, prior to cooling, pelleted into a cylinder like shape.

Himathongkham *et al*. [34] demonstrated that feed moisture and conditioning time were two factors that play a crucial role in the lethality of the pelleting process for bacteria. Most studies agree that the pelleting process is more effective at reducing *Salmonella* contamination. Cox *et al*. [24] reported that 92% of mash feed samples were positive for *Salmonella* but no pelleted samples were positive. However, Veldman *et al*. [18] found 21% of mash feeds and 1.4% of pelleted feeds were positive for *Salmonella*. Similarly, Jones *et al*. [19] found that 8.8% of mash feed samples and 4.2% of pelleted feed samples were contaminated with *Salmonella*.

If *Salmonella* is destroyed during the heat treatments, the possibility of re-contamination still exists. Raw feed ingredients can serve as a source of contamination to the plant environment and ultimately to the final feed product. Veldman *et al* [18] sampled raw feed ingredients and found 130 samples of fish meal (31%), 83 samples of meat and bone meal (4%), 58 samples of tapioca (2%) and 15 samples of maize grits (27%) were positive for *Salmonella*. The data presented by Jones *et al*. [19] indicated that dust within feed manufacturing facilities could serve as a major source of contamination to the final product. The authors suggested that mechanical vibrations and air currents around the pellet mill might have resulted in dust particles being dislodged and landing on the final pelleted feed. Davies and Wray [22] showed that the cooling unit was colonized by *Salmonella* which might serve as an airborne source of contamination to the final feed product. In addition, there is the possibility of feed being contaminated during transportation and/or storage [35]. With the possibilities of post-process contamination, detection methods are critical for preventing flock contamination. The next section will address current detection methods available and possibilities for future developments.

## 4. Detection

### 4.1. General Concepts

Detection of *Salmonella* in feed can be challenging due to low number of cells present in a large volume of feed. Riley [36] estimated a contamination rate of feed passing through a contaminated cooler would pick up 1 *Salmonella* organism per 10 to 100 tons if the facility was not receiving feed ingredient loads that were contaminated. At such a level of contamination, the challenge becomes designing both a sampling program and a method of detection that can detect 1 cell in 10 tons of feed.

Complications of isolating *Salmonella* from feed not only has been suggested to stem from the non-uniform distribution of the organism within the samples, but also from the effect of stress on the organisms from processing treatments used in feed mills [18,19,37]. In addition, the treatment of feed with formaldehyde can interfere with detection methods and give a false negative result [38].

Numerous detection methods have been developed for *Salmonella* such as culturing, immunological methods and nucleic acid based methods [38]. Typically, the method is chosen based on the application of the user. For example, if the desire is to not only detect but also to characterize *Salmonella*, the isolate will need to be recovered by culturing for further genotyping, antibiotic resistance typing, serotyping or other characterizations. However, if only presence or absence is necessary than nucleic acid detection assays are sufficient. Each method has advantages and disadvantages and every method has some limitations. The following sections will discuss the methods available and describe shortcomings and benefits for using a particular assay when applied to poultry breeder feeds.

### 4.2. Culturing

Traditional microbiological culture methods for the detection of *Salmonella* in feeds include selective enrichment and selective and/or differential plating. Culturing methods for the detection of *Salmonella* have been reviewed [39,40]. In general, plate agar media contains a pH indicator and lactose to differentiate *Salmonella* (a fermentor) from non-fermenting bacteria. Since most Salmonellae are hydrogen sulfide producers, tergetol can be added which results in *Samonella* colonies turning black. Other selective agents may be added such as antibiotics like novobicin which permit the growth of *Salmonella* while inhibiting competing microorganisms.

Chromogenic media work by using enzyme substrates that release colored dyes after hydrolysis, resulting in *Salmonella* colonies being colored and easily differentiated from other flora. This type of agar has an increased specificity over conventional media. However, some reports have shown that conventional media are less inhibitory and therefore more sensitive because stressed or injured microorganisms can be recovered [41,42]. Regardless of the plating media used, culturing methods are often considered as the “gold standards” but are time consuming in that they require days for results. Given that infection of an entire flock can occur in as few as three days [43], a method of detection that is more rapid than culturing is needed to implement control measures and control any further spread of infection.

### 4.3. Serology and Immunoassays

The genus of *Salmonella* consists of only two species, *S. enterica* and *S. bongori* but over 2,500 serotypes [44]. There are 47 possible *O*-antigens (lipopolysaccharide of cell wall) and 60 possible *H*-antigens (flagellum) with each serovar having its own unique combination of *O*- and *H*-antigens [45]. The name of each serovar was given based on the syndrome displayed, host specificity or geographical location [45].

Each serovar of *Salmonella* may vary widely in characteristics including severity of disease, virulence properties, ability to colonize chickens and survival in the environment outside the host [46–49]. For this reason, identification beyond the species level is necessary. Furthermore, serovar information is used by local and state health departments and CDC to monitor local, regional, and national trends of salmonellosis.

Serological based assays (immunoassays) such as ELISA have been widely used for *Salmonella* detection because they allow the sensitive and specific detection [50]. Immunological methods for the detection of *Salmonella* in animal feeds have been reviewed [40]. Immunoassays offer the ability to detect and distinguish serovars of *Salmonella.* However, immunoassays are hard to incorporate into an array format for multiple targets because high level of cross-reactivity between antibodies limits the number of targets that can be detected on the same array. Use of immunomagnetic beads (IMBs) in detection assays has been reported, and it shows great potential in *Salmonella* detection from complex matrices or environmental samples [51,52]. In IMB applications, however the number of target analytes detected in a single assay has been restricted to one or two due to high cross-reactivity and limited availability of commercial IMBs or antibodies for specific target organisms.

### 4.4. Nucleic Acid Detection

Polymerase Chain Reaction (PCR) has been gaining popularity as a tool in microbiological diagnosis due to the efficient, rapid and sensitive methods of detection. The methodology of PCR for the detection of foodborne pathogens has been reviewed previously [53]. Several variations of standard PCR, such as multiplex PCR and real-time PCR, have recently been employed for *Salmonella* detection, and these methods have provided high sensitivity with some assays being able to detect as few as 30 cells per sample [54]. The important criteria in the development of a nucleic acid based detection assay for *Salmonella* is the ability to detect all the diverse serotypes of the organism and PCR has been employed to replace conventional serotyping methods [55]. PCR-based serotypings depend on specific virulence genes, and have provided high specificity [56,57]. However there is a limitation on the number of target *Salmonella* serovars which can be detected in single PCR reaction. Even in multiplex PCR, it is difficult to incorporate more than five to six primer sets (correlating to five or six serovars) in one reaction due to cross-reactivity. Considering that there are at least 12 serotypes of *Salmonella* commonly associated with poultry [58], there is a clear need for an assay able to simultaneously detect multiple *Salmonella* serovars with minimal cross-reactivity.

### 4.5. Biosensors

Biosensors are being developed because they offer rapid and sensitive unconventional detection methods. Biosensors generally contain two components, a biological material (nucleic acid or antibody) closely associated with a transducing system. The transducer emits a signal when the target is captured that can be optical, electrochemical, thermometric, piezoelectric, magnetic or micromechanical. Biosensors differ from conventional detection methods because they are self-contained single units that have both the detection and reporting components.

Optical biosensors which utilize a fluorescent signal are typically the most common type of sensor [59]. However, biosensors using transducers other than optics have been developed for the specific detection of *Salmonella*. Olsen *et al*. [60] utilized bacteriophage specific for *Salmonella typhimurium*. In this biosensor, the capture of bacteria by bacteriophage that was adsorbed to a piezoelectric transducer resulted in a resonance frequency change measured with a Maxtek acoustical wave device. Su *et al*. [61] used an antibody bound to a gold coated quartz crystal surface with a gold electrode as a biosensor. After capturing *Salmonella*, changes in high-frequency impedance were directly correlated to the number of captured *Salmonella* cells. Pathirana *et al*. [62] and Kim *et al*. [63] also used a similar impedance analysis to create biosensors for the detection of *Salmonella typhimurium*.

DNA microarrays are a type of optical biosensors that detect hybridization of DNA sequences between bound probes having known sequences to fluorescently labeled DNA from an analyte. If hybridization is successful, a fluorescence signal is emitted upon excitation with a laser and the intensity of the signal can be used for quantification and identification of the analyte. Planar DNA microarrays allow thousands of specific DNA sequences to be screened simultaneously on a small single glass slide. Using DNA microarrays, multiple *Salmonella* serovars can be concurrently detected and the presence of virulence genes and antibiotic resistance genes can also be identified at the same time [64,65]. While planar microarrays offer the great potential for a rapid and sensitive detection of multiple pathogens [66,67] high fabrication cost and requirement for expensive equipments have been limiting their wide application in routine applications [68].

**Figure 2.**
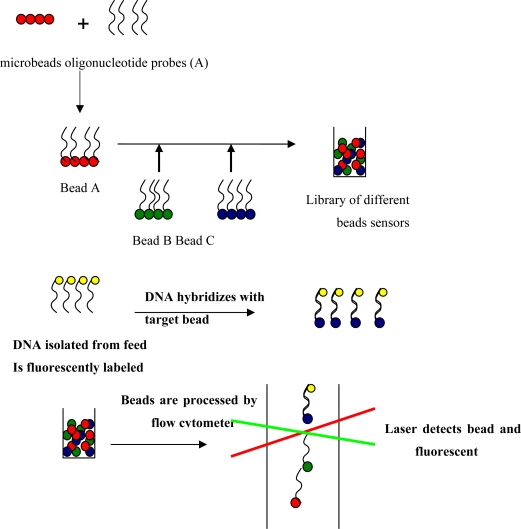
Illustrated description of bead based sensor array preparation, capturing of target and analysis. A, B and C denote different oligonucleotide probes (e.g. Bead A is beads functionalized with probe A). Red, green and blue colors indicate different types of encoding indicators.

Bead-based microarrays, as an alternative to planar microarrays, have been developed to perform multiplexed detection assays [68–70]. In bead-based arrays, microspheres are employed as solid support for the capture molecules (e.g., antibodies, oligonucleotide probes), instead of glass slides conventionally used in planar microarrays (Figure 2).

The individual microspheres are color-coded by distinct fluorescent dyes. In each DNA microarray, the oligonucleotide probe is immobilized to the surface of a distinct type of microspheres which are chemically functionalized. Different bead sets are then pooled to create a library, and hybridization is performed in a single vial or single well in a 96-well microplate containing the library of all bead types. After hybridization, presence of targets can be detected with a two-laser flow cytometer, where one laser interrogates the encoding dyes of beads to determine the probe identity and the other laser determines the presence of targets in the sample by reading the second fluorescent signal from hybridized targets.

Bead-based DNA arrays have several advantages over planar microarrays; (1) they can accommodate standard 96-well sample preparation systems; (2) since probes are coupled to distinct microspheres, each hybridization reaction can be analyzed; (3) if an additional target has to be included into the assay, a new type of probe-loaded bead can simply be added to the array unlike planar microarrays which require the new fabrication of arrays to add a target [68,69]. Bead-based arrays coupled with flow cytometry technology have been successfully applied for the simultaneous detection of multiple bacterial pathogens [71]; however this study was done with pure culture of target pathogens. Bead-based arrays have never been employed for detection of pathogens in more complex matrices such as feed or environmental samples. Bead-based arrays have been more commonly used in clinical applications such as simultaneous quantification of cytokines or autoantibodies from biological samples [72–75]. Bead-based arrays have the great potential for rapid and sensitive identification of *Salmonella* from feed. The criteria for optimal bead based array design are listed in Table 1.

An initial step for rapid microarray development involves the selection of target genes and design of probes and primers that detect and characterize *Salmonella* spp. which are commonly found in poultry breeder feeds. However, effective sample preparation methods to minimize the effect of environmental factors are usually required to retrieve *Salmonella* from feed matrices. Immunomagnetic separation using anti-*Salmonella* magnetic beads can be employed as a standard method to separate *Salmonella* from feed matrices [76]. Cultural pre-enrichment also can be utilized to optimize sample preparation to alleviate any inhibitory effect from feed matrices while keeping the total assay time short.

Both optical and electrochemical biosensors offer advantages, but also come with disadvantages. Optical techniques have been demonstrated to provide better sensitivity than electrochemical ones [59]. Electrochemical techniques offer simplicity over optical detection methods. However, optical techniques offer the ability to capture and detect many targets and for this reason are usually more costly. Some biosensors are sensitive but they still are not capable of the same detection levels as traditional techniques.

## 5. Future prospects

Sweden implemented a Hazard Analysis and Critical Control Point (HACCP) program for animal feed in 1991 and since then a decline in the annual incidence of domestically acquired human salmonellosis has been observed, with a drop from 14 cases per 100,000 population in 1991 to 8 cases per 100,000 population in 2000 [77]. Under the Swedish HACCP program, approximately 7,000 samples from feed mills are analyzed annually, of which 40% are obtained before heat treatment. Detection of any positive samples generates more sampling and corrective actions are taken. Sweden has an integrated surveillance of feed, animals, food, and humans which allows investigators to track trends and monitor the impact of interventions and has virtually eliminated *S. enterica* from domestically produced animal feed and red and white meat [77,78].

Jones *et al*., [7] also underlined the need for a comprehensive approach in the control of *Salmonella* contamination in the broiler production and processing system. As a follow up to this point, Jones and Ricke [79] outlined a specific Hazard Analysis of Critical Control Points approach for the control of *Salmonella* in feeds. Presently, a farm or feed mill in the U.S. may adopt several good manufacturing practices (GMPs) to reduce feed recontamination. Feed bins, feed pans, cross augers, hoppers, silos and transport trucks and silos could be regularly cleaned and painted with ceramic paint to prevent the buildup of caked feed that may be contaminated with pathogenic molds, bacteria, or mycotoxins [80,81]. Systems have been in development which may disinfect truck tires while the truck is still moving, reducing soil contamination between the farm and feed mill [82]. Dust in feed mills may be sampled for airborne *Salmonella* spp., a general indication of *Salmonella* spp. presence in the environment that may reduce the problem of sample size [83]. Monitoring *Salmonella* spp. either in feed mixtures or feed ingredients will probably require some sort of direct detection of *Salmonella* spp. Improvements in detection methods that are more sensitive and rapid are needed to control *Salmonella* from a top down approach. Because only a few cells of *Salmonella* can infect a chick, sensitivity of an assay is crucial. Furthermore, given the rapid transmission of *Salmonella* within a flock, an assay that could be performed in less than 24 hours would give producers time to implement corrective actions and control transmission.

## 6. Conclusions

With an increase in consumption of animal derived foods, the number of foodborne illnesses associated with poultry has also increased in recent years. Safety of poultry will be greatly improved by rapid and sensitive pathogen detection system. Development of bead-based DNA microarray coupled with flow cytometry and PCR amplification would be ideal for simultaneous detection and differentiation of *Salmonella* serovars commonly associated with poultry breeder feed contamination. The advantages of a DNA microarray include simplicity, reusability, and multiplexing capability, and would make it cost-effective and sensitive technology. This highly practical technology can readily be applied to other types of feeds and feed ingredients as well.

## Figures and Tables

**Table 1.** Criteria for optimal DNA bead-based microarray detection.

Target genes and designed probe and primer sequences can detect target *Salmonella* serovars with minimal cross-reactivity. Bead-based DNA microarrays can simultaneously detect multiple serovars using a 96-well microplate format. Developed bead-based microarrays will work with both synthetic and culture samples. Microarrays can detect *Salmonella* pathogens in various feed and feed ingredient samples from both experimentally and naturally contaminated samples.
